# p38α and p38β regulate osmostress-induced apoptosis

**DOI:** 10.1016/j.jbc.2024.108061

**Published:** 2024-12-07

**Authors:** Nabil Ben Messaoud, José M. López

**Affiliations:** Institut de Neurociències, Departament de Bioquímica i Biologia Molecular, Unitat de Bioquímica, Facultad de Medicina, Universitat Autònoma de Barcelona, Cerdanyola del Vallès, Barcelona, Spain

**Keywords:** apoptosis, p38, stress, oocyte, *Xenopus*, cell death, caspase, osmotic shock

## Abstract

Hyperosmotic shock induces cytochrome c release and caspase-3 activation in *Xenopus* oocytes. Different signaling pathways engaged by osmostress converge on the mitochondria to trigger cell death. The mitogen-activated protein kinases (MAPKs) JNK1-1 and JNK1-2 are early activated by hyperosmotic shock and sustained activation of both isoforms accelerates the apoptotic program. Indeed, sustained activation of p38 accelerates osmostress-induced cell death, but the p38 isoforms involved are not well characterized. Here we study the expression and activation of *Xenopus* p38 isoforms in response to hyperosmotic stress. We find that p38α, p38β, and p38γ are early activated by hyperosmotic shock and sustained activation of p38α and p38β accelerates osmostress-induced apoptosis. Moreover, microinjection of cytochrome c in the oocytes induces caspase-3 activation and p38α and p38β phosphorylation suggesting that caspases and kinases are interlinked in a positive feedback loop to promote cell death. In summary, we present a more complete view of the mechanisms involved in osmostress-induced apoptosis.

Hyperosmotic shock has many damaging effects on cells by promoting water flux out of the cell, causing cell shrinkage and intracellular dehydration (1). Cells respond to osmostress with compensatory molecular adaptations that allow them to restore homeostasis and cellular function (2). However, when cells are no longer able to compensate for hyperosmotic stress and the amount of damage is too great, they trigger apoptosis (3, 4, 5, 6, 7).

We have reported that hyperosmotic shock induces cytochrome c release and caspase-3 activation in *Xenopus* oocytes (8). Several mechanisms regulate osmostress-induced apoptosis in this cellular system. Hyperosmotic shock induces rapid calpain activation and high levels of Smac/DIABLO release from the mitochondria (early events) before significant amounts of cytochrome c are released to promote caspase-3 activation (late events) (9). We also studied the role of Bcl-2 family members on osmostress-induced apoptosis. Bid is early proteolyzed in small amounts, by a yet not identified protease/caspase, contributing to cytochrome c release, and later on high amounts are proteolyzed by caspase-3 creating a positive feedback loop (10). Hyperosmotic shock also activates very quickly the MAPKs (mitogen-activated protein kinases) p38 and JNK (8, 9, 10, 11). Simultaneous inhibition of both pathways, adding SB203580 plus SP600125, reduces osmostress-induced apoptosis (9). Accordingly, sustained activation of p38 (9) or JNK (10) accelerates the release of cytochrome c and caspase-3 activation. We concluded from these studies that different pathways, early induced by osmostress, converge on the mitochondria to trigger apoptosis (9, 10, 12).

We also characterized the JNK isoforms activated by hyperosmotic shock and their role in apoptosis. JNK1-1 and JNK1-2 are early activated by osmostress and sustained activation of both isoforms accelerates the apoptotic program. Moreover, when caspase-3 is activated, JNK1-2 is proteolyzed at Asp385, increasing the release of cytochrome c and caspase-3 activity, thus creating another positive feedback loop (10). Although p38 is involved in the apoptotic process, it is not clear which specific p38 isoforms are early activated by osmostress and how they regulate cell death. Interestingly, we reported that cytochrome c injection in *Xenopus* oocytes induces phosphorylation of p38 through caspase-3 activation, suggesting an additional positive feedback loop (11).

The p38 MAPK family is composed of four isoforms that have 60 to 70% homology between them. p38α (MAPK14), p38β (MAPK11), p38γ (MAPK12), and p38δ (MAPK13) are encoded by different genes and have different expression patterns in tissues, with p38α being the major component, expressed ubiquitously in most cell types, while the other isoforms are expressed specifically in different tissues. p38β is preferentially expressed in the brain, p38γ in skeletal muscle, and p38δ in endocrine glands (13). p38 MAPKs are activated by dual phosphorylation of tyrosine and threonine residues in a conserved Thr-Gly-Tyr motif, in the activation loop, by MKK3 and MKK6 (14, 15, 16). In some circumstances, such as ultraviolet radiation, MKK4, an activator of JNK, may contribute to p38 activation (17). Hyperosmotic shock has been described to activate MKK6 and MKK3 in mouse embryonic fibroblasts (18). MKK6 activates p38α, p38β, and p38γ in response to hyperosmotic shock, but does not activate so well p38δ, while MKK3 activates the four isoforms, being the main activator of p38δ (18). Autophosphorylation may also contribute to the activation of p38 MAPKs (19, 20).

p38 activation can have a pro- or an anti-apoptotic function depending upon the stimuli and the cellular context (12, 21). It seems clear that early transient activation of p38 promotes cell survival, whereas prolonged activation mediates cell growth arrest or apoptosis (12, 22, 23, 24, 25). In human erythrocytes, p38 activation favors hyperosmotic shock-induced apoptosis (26), although it is not clear which isoforms are relevant.

Little is known about the function of p38 in *Xenopus* oocytes. p38α and p38γ have been cloned and expressed in the oocytes and both isoforms are activated by a constitutively active MKK6 mutant (MKK6-DD) (27, 28, 29). It has been described that progesterone activates p38γ and regulates G2/M transition during meiotic progression (29). However, nothing is known about the role of the different p38 isoforms in osmostress-induced apoptosis.

Here we study the expression and activation of p38 isoforms in *Xenopus* oocytes exposed to hyperosmotic stress. We find that p38α, p38β, and p38γ are early activated by hyperosmotic shock and sustained activation of p38α and p38β accelerates osmostress-induced apoptosis. Moreover, cytochrome c injection in *Xenopus* oocytes induces caspase-3 activation and p38α and p38β phosphorylation indicating that caspases and kinases are linked in a positive feedback loop to promote cell death.

## Results

### *Xenopus* p38α and p38γ are phosphorylated in response to hyperosmotic shock

The expression of p38 isoforms was analyzed in *Xenopus laevis* oocytes by RT-PCR. PCR products were obtained for p38α, p38β, and p38γ, but not for p38δ (Fig. S1), suggesting that p38δ is not expressed in the oocytes.

**Figure 1:**
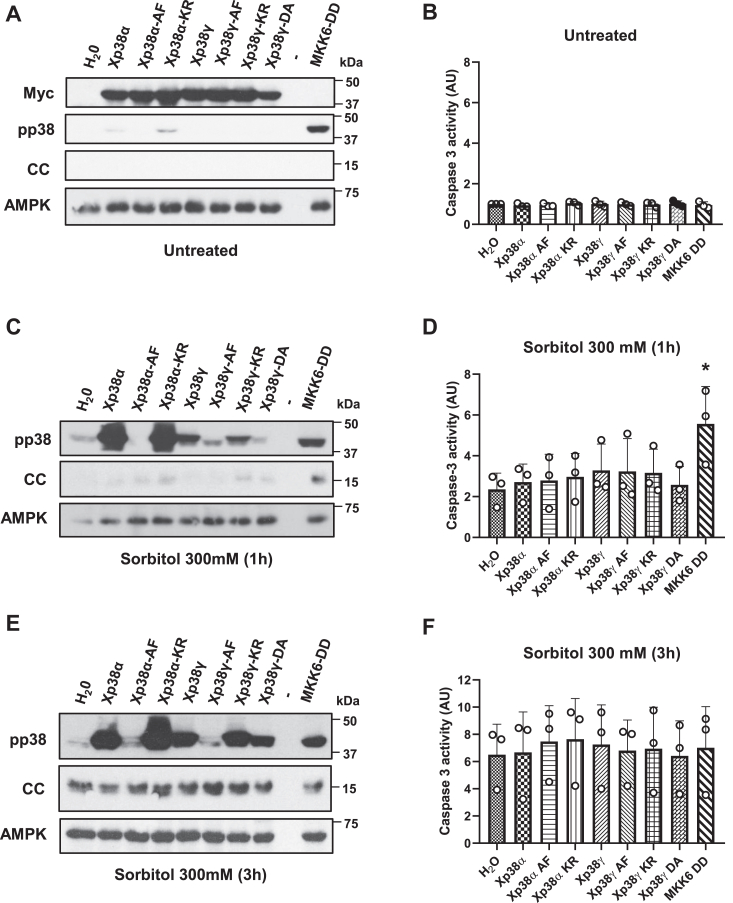
**Xp38α and Xp38γ are activated by hyperosmotic shock.***A, C*, and *E*, expression and phosphorylation of Xp38α, Xp38γ, and their mutants. Oocytes were injected with 50 nl of H_2_O or cRNAs (5 ng in 50 nl) Xp38α, Xp38α-AF, Xp38α-KR, Xp38γ, Xp38γ-DA, or MKK6-DD and 18 h later exposed to osmotic shock (300 mM sorbitol) for 1 h, 3 h, or non-treated. Expression of p38 isoforms was confirmed with Myc antibodies (*A*). MKK6-DD is a constitutively active mutant without Myc tag. pp38 and cytosolic cytochrome c (CC) levels were analyzed by Western blot and AMPK was used as a loading control. *B*, *D*, and *F*, caspase-3 activity was measured in all the conditions assayed, giving value 1 to non-treated water-injected oocytes. Expression of Xp38α, Xp38γ, or their inactive mutants did not modify hyperosmotic shock-induced apoptosis. Results in panels (*B*, *D*, and *F*) are the mean ± SD of three independent experiments. ∗*p* < 0.05 compared to water-injected oocytes (ANOVA and Dunnett’s test). Western blots are representative of three independent experiments (see Figs. S2 and S3 for additional experiments and blots quantification).

*Xenopus* oocytes were microinjected with cRNAs corresponding to the wild type p38α (Xp38α), p38γ (Xp38γ), or their catalytically inactive mutants (-KR, -AF, -DA, see Experimental Procedures section). All Xp38 isoforms have a Myc epitope at the amino-terminal end allowing their detection by Western blot. Oocytes injected with the different cRNAs expressed similar levels of the corresponding proteins (Figs. 1*A* and S2, *A* and *D*) and did not show any significant change in caspase-3 activity (Fig. 1*B*) or cytochrome c release (Figs. 1*A* and S3*D*) compared to water-injected oocytes. Oocytes expressing a constitutively active MKK6 (MKK6-DD), the kinase upstream of p38, showed marked phosphorylation of p38 (Figs. 1*A* and S3*A*) but not an increase in caspase-3 activity (Fig. 1*B*). Treatment of oocytes with 300 mM sorbitol for 1 h and 3 h induced the phosphorylation of endogenous p38 at Thr181 and Tyr183, a large increase of phosphorylation in oocytes expressing Xp38α or Xp38α-KR, and a significant increase in those expressing Xp38γ, Xp38γ-KR or Xp38γ-DA (Figs. 1, *C* and *E*, S2, and S3). It is well known that the overall activity of p38 is correlated with the dual phosphorylation of Thr181 and Tyr183 (30). As expected, the mutants Xp38α-AF and Xp38γ-AF, with residues Thr and Tyr mutated to Ala and Phe, were not phosphorylated in the oocytes treated with sorbitol (Figs. 1, *C* and *E*, S2, and S3). There were no significant changes in caspase-3 activity and cytochrome c release between water-injected oocytes and those expressing Xp38α, Xp38γ isoforms, or their mutants (Figs. 1, *D* and *F* and S3, *E* and *F*). As reported previously, expression of MKK6-DD accelerated caspase-3 activation and cytochrome c release in oocytes exposed to hyperosmotic shock (Fig. 1*D*) (9).

### *Xenopus* p38β is phosphorylated in response to hyperosmotic shock

**Figure 2:**
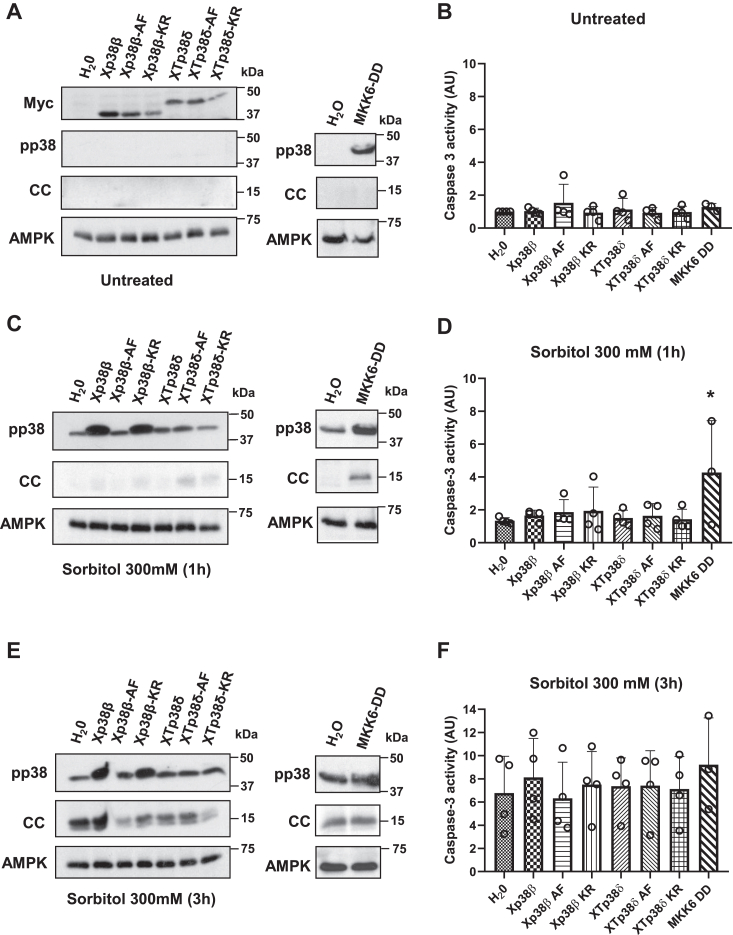
**Xp38β is activated by hyperosmotic shock.***A, C*, and *E*, expression and phosphorylation of p38 isoforms and mutants. Oocytes were injected with 50 nl of H_2_O or cRNAs (5 ng in 50 nl) Xp38β, Xp38β-AF, Xp38β-KR, Xp38δ, Xp38δ-AF, Xp38δ-KR, or MKK6-DD and 18 h later exposed to osmotic shock (300 mM sorbitol) for 1 h, 3 h, or non-treated. Expression of p38 isoforms was confirmed with Myc antibodies (A). pp38 and cytosolic cytochrome c (CC) levels were analyzed by Western blot and AMPK was used as a loading control. *B, D*, and *F*, caspase-3 activity was measured in all the conditions assayed, giving value 1 to non-treated water-injected oocytes. Expression Xp38β, Xp38δ, or their inactive mutants did not modify osmostress-induced apoptosis. Data in (*B*, *D*, and *F*) are represented as mean ± SD of three or four independent experiments. ∗*p* < 0.05 compared to water-injected oocytes (ANOVA and Dunnett’s test). Western blots are representative of three independent experiments (see Figs. S4 and S5 for additional experiments and blots quantification).

*X. laevis* p38β (Xp38β), *Xenopus tropicalis* p38δ (XTp38δ), or their respective mutants -AF and -KR, were expressed in *Xenopus* oocytes (Figs. 2*A*, and S4, *A*, and *D*). Treatment with 300 mM sorbitol induced phosphorylation of Xp38β and Xp38β-KR, but not Xp38β-AF (Figs. 2, *C* and *E* and S4, and S5). XTp38δ was not phosphorylated in response to osmostress in some experiments (Fig. 2, *C* and *E*), but a slight phosphorylation of XTp38δ and XTp38δ-KR was detected in others (Fig. S4, *B*, *C*, *E*, and *F*). Expression of Xp38β, XTp38δ, or their mutants, did not alter the levels of caspase-3 activity induced by osmostress compared to water-injected oocytes (Fig. 2, *B*, *D*, and *F*). As described before, oocytes expressing MKK6-DD presented increased caspase-3 activity compared to water-injected oocytes (Fig. 2*D*).

Since XTp38δ was not well phosphorylated in response to osmostress, we also studied the response of human p38δ (Hp38δ). As shown in Fig. S6*A*, hyperosmotic shock-induced phosphorylation of Hp38δ, but to a lesser extent than Xp38β. Hp38δ-AF and Hp38δ-KR mutants were not significantly phosphorylated by sorbitol treatment (Fig. S6*B*). Expression of wild-type Hp38δ or their mutants did not modify caspase-3 activity induced by hyperosmotic shock (Fig. S6, *C*–*F*).

In summary, the abovementioned results indicate that hyperosmotic shock induces phosphorylation of Xp38α, Xp38β, and Xp38γ in *Xenopus* oocytes, and that expression of these isoforms, or their mutants, does not modify osmostress-induced apoptosis.

### A constitutively active Xp38α or Xp38β accelerates osmostress-induced apoptosis

It is clear from the previous results that a constitutively active MKK6 accelerates hyperosmotic shock-induced apoptosis (Figs. 1 and 2). To determine which p38 isoform/s is/are involved in this process, we expressed a constitutively active p38α (Xp38α-CA), a constitutively active p38β (Xp38β-DA/YL), or both (see Experimental procedures section for details). These mutants contain a Myc sequence at the N-terminal end that allows us to verify their expression levels by Western blot.

**Figure 3:**
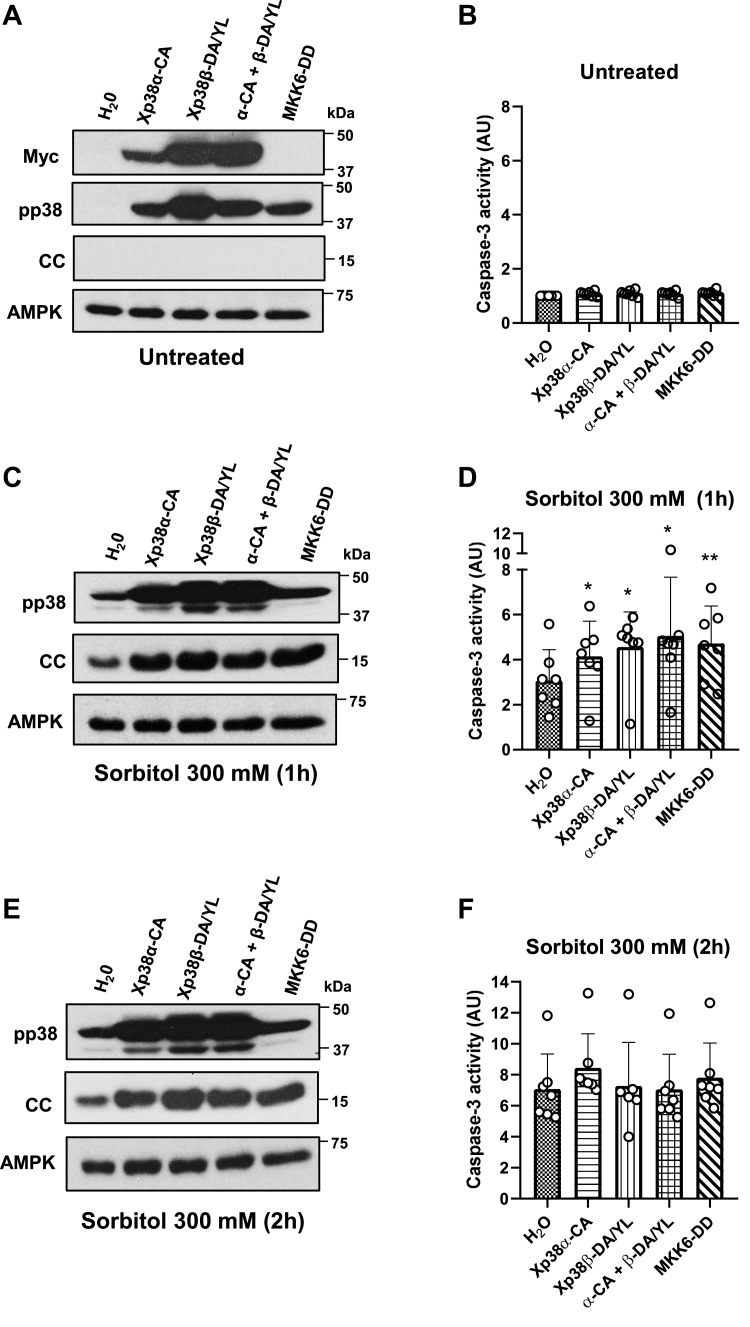
**Constitutively active Xp38α (CA) or Xp38β (DA/YL) expression accelerates hyperosmotic shock-induced apoptosis.***A*, *C*, and *E*, oocytes were injected with 50 nl of H_2_O or cRNAs (5 ng in 50 nl) Xp38α-CA, Xp38β-DA/YL, a combination of both, or MKK6-DD and 18 h later exposed to osmotic shock (300 mM sorbitol) for 1 h, 2 h, or non-treated. Expression of p38 mutants was confirmed with Myc antibodies (A). pp38 and cytosolic cytochrome c (CC) levels were analyzed by Western blot and AMPK was used as a loading control. *B, D*, and *F*, caspase-3 activity was measured in all the conditions assayed, giving value 1 to non-treated water-injected oocytes. Results represent the mean ± SD of seven independent experiments. ∗*p* < 0.05, ∗∗*p* < 0.01 compared to water-injected oocytes (ANOVA and Dunnett’s test). Western blots are representative of three independent experiments (see Figs. S7 and S8 for additional experiments and blots quantification).

Xp38-CA or Xp38-DA/YL were efficiently expressed in the oocytes, inducing a marked phosphorylation of p38, but not cytochrome c release or caspase-3 activation, like MKK6-DD (Figs. 3, *A* and *B*, S7, and S8). These results indicate that sustained activation of Xp38α, Xp38β, or both, is not sufficient to induce apoptosis in untreated oocytes. However, treatment with 300 mM sorbitol for 1 h significantly increased caspase-3 activity in the oocytes expressing Xp38-CA, Xp38-DA/YL, or both, compared to water-injected oocytes (Fig. 3*D*), and was correlated with a massive release of cytochrome c (Figs. 3*C*, S7, *B* and *E*, and S8*E*). As expected, MKK6-DD also increased caspase-3 activity and cytochrome release relative to water-injected oocytes (Figs. 3, *C* and *D*, S8*E*).

Very high levels of caspase-3 activity were reached 2 h after sorbitol treatment, with no significant differences observed between the conditions analyzed (Fig. 3*F*).

These results suggest that sustained activation of Xp38α and Xp38β isoforms has a pro-apoptotic role in hyperosmotic shock-induced apoptosis.

### Kinases and caspases are linked in a positive feedback loop: cytochrome c induces caspase-3 activation and phosphorylation of Xp38α and Xp38β

As we reported previously, the injection of cytochrome c in *Xenopus* oocytes induces rapid caspase-3 activation (30 min) and p38 phosphorylation (1 h) (11). This effect was blocked by a caspase-3 inhibitor, demonstrating that p38 phosphorylation was caspase-3 dependent (11). These results suggested that p38 was engaged in a positive feedback loop.

**Figure 4:**
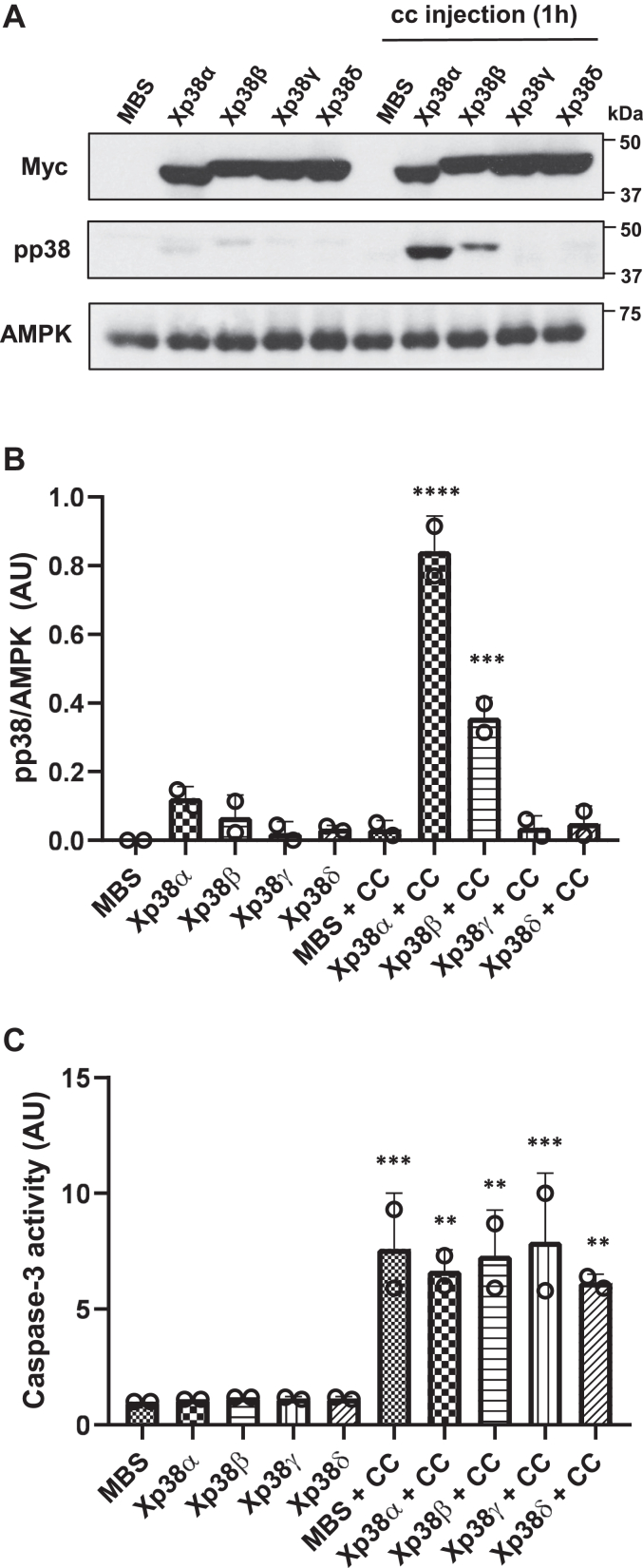
**Cytochrome c injection in *Xenopus* oocytes induces caspase-3 activation and p38α and p38β phosphorylation.***A*, oocytes were injected with 50 nl of MBS or cRNAs (5 ng in 50 nl), Xp38α, Xp38β, Xp38γ, or Xp38δ and 18 h later injected with MBS (as a control) or horse cytochrome c (CC) (0.5 μM final concentration) dissolved in MBS and pools of 20 oocytes were lysed 1 h later to analyze Myc, pp38, and AMPK by Western blot. A representative blot of two independent experiments is shown (see Fig. S9 for additional experiment). *B*, blots were quantified with Image J and the ratio pp38/AMPK represented. Results are the mean ± SD of two independent experiments. ∗∗∗*p* < 0.001, ∗∗∗∗*p* < 0.0001 compared to oocytes of the same condition but non-injected with cytochrome c (ANOVA and Fisher’s LSD test). *C*, caspase-3 activity was determined in all the extracts and represented as arbitrary units (AU), giving value 1 to oocytes injected with MBS. Results are the mean ± SD of two independent experiments. ∗∗*p* < 0.01, ∗∗∗*p* < 0.001 compared to oocytes of the same condition but non-injected with cytochrome c (ANOVA and Fisher’s LSD test).

**Figure 5:**
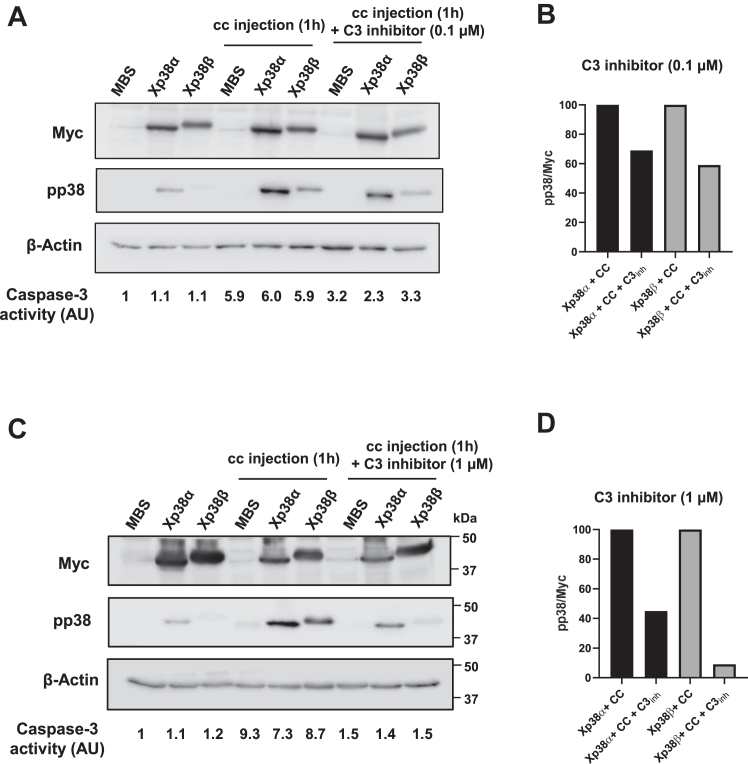
**Caspase-3 inhibition reduces phosphorylation of p38α and p38β induced by cytochrome c injection.***A*, oocytes were injected with MBS, horse cytochrome c (CC) (0.5 μM final intracellular concentration), or CC (0.5 μM) plus caspase-3 inhibitor Ac-DEVD-CHO (0.1 μM final intracellular concentration) and pools of 20 oocytes were lysed 1 h later to analyze Myc, pp38, and β-Actin by Western blot. Caspase-3 activity was determined as previously described (see values at the bottom of the blot). *B*, pp38 and Myc levels were quantified from the blot with Image J and the ratio pp38/Myc represented in arbitrary units (AU), giving value 100% to oocytes expressing Xp38α or Xp38β injected with cytochrome C. *C* and *D*, oocytes were treated and analyzed as described above, but Ac-DEVD-CHO was injected at 1 μM final concentration. Note that caspase-3 activity was markedly reduced in *C* compared with *B*.

To clarify the p38 isoforms involved in this loop, each isoform was expressed in *Xenopus* oocytes and subsequently injected with cytochrome c. Phosho-p38 levels were analyzed by Western blot 1 hour after cytochrome c injection. As shown in Figures 4, *A* and *B* and S9, all the p38 isoforms were expressed at similar levels in the oocytes, but only Xp38α and Xp38β were phosphorylated 1 h after cytochrome c injection. As expected, caspase-3 activity was increased in the oocytes injected with cytochrome c (Fig. 4*C*). We can rule out a nonspecific stress response due to the exogenous protein injection since we have previously shown that cytochrome c from yeast (*Saccharomyces cerevisiae*), which cannot trigger caspase-3 activation in *Xenopus* oocytes (31), does not induce p38 phosphorylation (11). Importantly, injection of a specific caspase-3 inhibitor (Ac-DEVD-CHO) in the oocytes at 0.1 μM (final concentration) decreased caspase-3 activity and phosphorylation of Xp38α and Xp38β induced by cytochrome c (Fig. 5, *A* and *B*). A higher concentration of the inhibitor (1 μM) markedly decreased caspase-3 activity as well as Xp38α and Xp38β phosphorylation (Fig. 5, *C* and *D*). In summary, our results suggest that sustained activation of Xp38α and Xp38β accelerates cytochrome c release (Fig. 3), which in turn induces caspase-3 activation and phosphorylation of Xp38α and Xp38β (Fig. 4), thus creating a positive feedback loop.

## Discussion

*Xenopus* oocytes have several advantages in studying cell death mechanisms. It is a very good system for expressing wild-type and mutant proteins. Since *Xenopus* oocytes at stage VI are transcriptionally inactive, all the effects observed are non-genomic. Cytochrome c release and protein kinase activation can be measured in single cells by Western blot (8, 11). One of the most important advantages of *Xenopus* oocytes is that cytochrome c can be injected in the cells to investigate the signaling pathways activated downstream, independently of the initial stimuli. This facilitates the study of positive feedback loops engaged during cell death. Hyperosmotic shock induces apoptosis in *Xenopus* oocytes through activation of different signaling pathways (9). In previous work, we described the basic properties of p38 in response to hyperosmotic shock (11). Xp38 is quickly activated (5 min) with an ultrasensitive and sustained response (11). When maximum p38 activation is attained, cytochrome c is released from the mitochondria to the cytosol, promoting caspase-3 activation. We also demonstrated that sustained activation of p38 accelerates cytochrome-c release and caspase-3 activation (9) and, interestingly, that cytochrome c release induces p38-phosphorylation in a caspase-3 dependent manner, suggesting a positive feedback loop (11). However, it was not clear in the previous studies which p38 isoforms are involved in this process.

Here, we show that Xp38α, Xp38β, and Xp38γ are detected by RT-PCR in *X. laevis* oocytes (Fig. S1). Ectopic expression of different isoforms in the oocytes allows studying their activation in response to osmostress. The three isoforms are activated by hyperosmotic shock (Figs. 1 and 2). Xp38δ was not detected by RT-PCR, and *X. tropicalis* p38δ (XTp38δ) ectopically expressed in the oocytes was weakly phosphorylated in response to hyperosmotic shock. Likewise, human p38δ (Hp38δ) expressed in oocytes was phosphorylated in response to osmostress but to a lesser extent than Xp38β. Based on these results, we consider that endogenous Xp38δ is not expressed in oocytes. Even if expressed at low levels, it would hardly be activated by osmostress.

MAPKs have a dual role in apoptosis, since they act as activators or inhibitors, depending on the cell type and the stimulus (12). However, it seems clear that the kinetics and the intensity of the signaling pathway is going to be important for cell fate. Sustained activation of MAPKs usually favors caspase-3 activation, which in turn engages several positive feedback loops promoting cell death (12, 32).

In this study we show that expression of a constitutively active Xp38α or Xp38β accelerates osmostress-induced apoptosis in *Xenopus* oocytes (Fig. 3). Therefore, activation of both isoforms by hyperosmotic shock regulates the apoptotic program.

Xp38γ is also activated by osmostress (Fig. 1) but its role in apoptosis is uncertain. We could not obtain a constitutively active Xp38γ, but it has been reported that sustained activation of this isoform induces meiotic progression in *Xenopus* oocytes (29). Indeed, we observed that co-expression of MKK6-DD and Xp38γ induced germinal vesicle breakdown (GVBD) in the oocytes, detected as the appearance of a white spot in the animal pole (data not shown). It has been reported that Xp38γ induces meiotic progression through activation of the phosphatase Cdc25C (29). More studies are necessary to address the role of p38γ in osmostress-induced apoptosis.

Importantly, we show that cytochrome c injection in the oocytes induces phosphorylation of Xp38α and Xp38β, but not Xp38γ or XTp38δ (Fig. 4). Therefore, our results imply that p38 is activated by two different inputs at different times: First, hyperosmotic shock induces sustained activation of Xp38α, Xp38β (probably mediated by activation of MKK6) that accelerates the apoptotic program. Second, the confluence of different signaling pathways in the mitochondria promotes cytochrome c release and caspase-3 activation, which in turn activates Xp38α and Xp38β creating a positive feedback loop. This loop, in combination with others engaged by caspase-3 (12), would make irreversible the apoptotic program.

How caspase-3 activation induces phosphorylation of p38α and p38β? It has been reported that caspase-3 induces proteolysis and constitutive activation of MEKK1 (33, 34), which in turn activates JNK and p38 (35). However, cytochrome c injection did not induce rapid phosphorylation of JNK (data not shown), thus discarding proteolysis and constitutive activation of MEKK1. Another possibility is that caspase-3 activation would increase the levels of reactive oxygen species (ROS) through disruption of the functions of complex I and II of the electron transport chain (36), which in turn would activate p38 through activation of MINK and/or ASK1 (37, 38). Sustained activation of p38 also induces important metabolic changes and enhances the respiration rate, increasing the production of mitochondrial ROS, which contributes to p38-induced apoptosis (39). More studies are necessary to characterize the signaling pathway that induces Xp38α and Xp38β phosphorylation mediated by caspase-3 activation.

Sensing environmental changes in salinity and activation of p38 is an ancient function conserved in both unicellular organisms and animals (40, 41). Although cells have developed mechanisms to adapt to osmotic changes and survive, when the stress is intense or persistent the cellular machinery initiates a death program.

**Figure 6:**
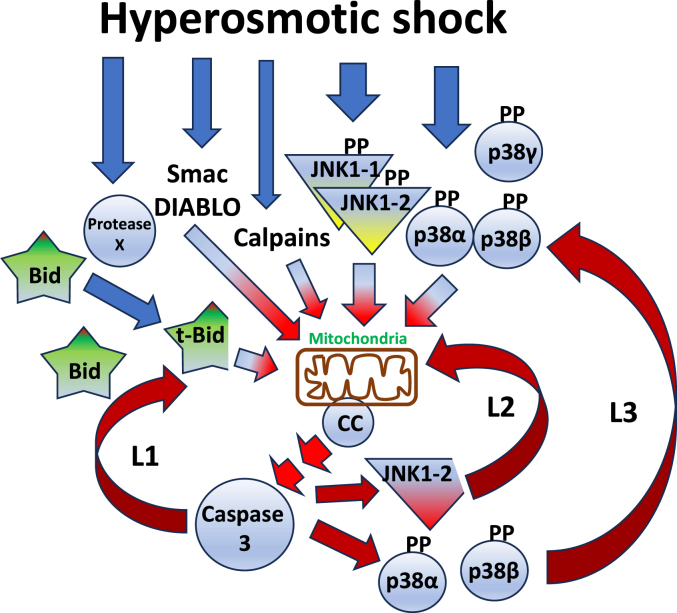
**Model for osmostress-induced apoptosis.** Like a heavy rain, hyperosmotic shock rapidly activates several signaling pathways that converge in the mitochondria to increase their permeability releasing cytochrome c to the cytosol. In *Xenopus* oocytes, hyperosmotic shock induces rapid calpain activation, Smac/DIABLO release from the mitochondria, cleavage of small amounts of Bid by an unknown protease, and activation of JNK1-1, JNK1-2, p38α, p38β, and p38γ. Sustained activation of JNK1-1, JNK1-2, p38α, and p38β in combination with Smac/DIABLO, t-Bid, and calpains converge on the mitochondria to induce the release of cytochrome c into the cytosol, which in turn activates caspase-3. The activation of caspase-3 induces JNK1-2 and Bid proteolysis, as well as p38α and p38β phosphorylation. These events, in turn, promote additional cytochrome c release and caspase-3 activation engaging at least three positive feedback loops (L1-L3) to complete the cell death program.

In Figure 6, we present a model for osmostress-induced apoptosis, which summarizes our knowledge about this process. The apoptotic program is characterized by two different phases: In an *early phase*, hyperosmotic shock induces synchronic activation of different pathways: calpains, Bid cleavage (by an unknown protease), MAPKs phosphorylation (JNK1-1, JNK1-2, p38α, p38β, p38γ) and release of Smac/DIABLO. We propose that this early phase is reversible, with crosstalk of anti-apoptotic and pro-apoptotic signals. In this phase, the oocytes could recover if the stress is removed or weakened. MAPKs (JNK and p38 isoforms) are sensors of stress that could activate early anti-apoptotic substrates, but strong and/or sustained activation of MAPKs would activate late pro-apoptotic substrates. These substrates, not yet characterized, could include different Bcl-2 family members. We know that Bcl-X overexpression blocks osmostress-induced apoptosis (10). In a *late phase*, the different signaling pathways converging on the mitochondria trigger cytochrome c release and caspase-3 activation. Caspase-3 induces JNK1-2 proteolysis at Asp385 and massive proteolysis of Bid at Asp52, which in turn induces more cytochrome c release and caspase-3 activation engaging two positive feedback loops (10). Our previous results also indicate that caspase-3 increases Smac/DIABLO release (9). Here we show that caspase-3 induces phosphorylation and activation of p38α and p38β. In addition, it has been reported that caspases can induce proteolysis of the calpain inhibitor calpastatin (42, 43), thus increasing calpain activation in a positive loop. Therefore, hyperosmotic shock induces different signaling pathways that converge on the mitochondria to engage an irreversible apoptotic program through caspase-3-dependent activation of multiple positive feedback loops.

In summary, we have now a panoramic view of the signaling pathways and feedback loops activated by osmostress that regulate apoptosis. However, much work remains to be done to understand this complex biological process. We know that sustained activation of several protein kinases (p38α, p38β, JNK1-1, and JNK1-2) are pro-apoptotic, opening new avenues of research to identify their targets. A complete understanding of osmostress should also consider transient anti-apoptotic mechanisms engaged before cell death. Deciphering the mechanisms of osmostress-induced apoptosis will give insights in how apoptotic programs are regulated and could be useful in understanding some human illnesses caused by alterations in fluid osmolarity.

## Experimental procedures

### Oocyte isolation and treatment

Oocytes were obtained from sexually mature *X. laevis* females (purchased from Centre d’Elevage de Xenopes, Montpellier, or from *Xenopus* Express), anesthetized in 0.02% benzocaine and portions of ovary were removed through a small incision on the abdomen. The incision was sutured, and the animal was returned to a separate tank until it had fully recovered from the anesthesia. It was then returned to a large tank in which all the frogs were kept for at least 4 weeks until the next surgery. The protocol was approved by the Committee on the Ethics of Animal Experiments of the Universitat Autònoma de Barcelona (Permit Number: CEEAH 439) and all efforts were made to minimize animal suffering. The tissue was dissected into small pieces, if the ovaries were healthy enough to use, and oocytes were defolliculated for 2 to 3 h at room temperature with collagenase/dispase (0.8 mg/ml (Sigma), 0.48 mg/ml (Roche)) in Modified Barth’s Saline (MBS: 5 mM HEPES, 88 mM NaCl, 1 mM KCl, 1 mM MgSO4·7H_2_O, 2.5 mM NaHCO_3_, 0.7 mM CaCl_2_, pH 7.8) with gentle agitation. The defolliculated oocytes were then washed thoroughly with MBS and transferred to Petri dishes. Stage VI oocytes were sorted manually and incubated overnight in MBS at 18 °C. The next day, healthy survivors were selected and transferred to a Petri dish containing fresh MBS.

### RT-PCR and DNA constructs

Total RNA was isolated from *X. laevis* oocytes (stage VI) with the method described by McGrew (44) and kept at −70 °C. First-strand cDNA was synthesized with RevertAid M-MuLV Reverse Transcriptase (Fermentas) in a 20 μl reaction using 250 ng of total RNA and poly (dT) primer. The reaction mixture was incubated for 1 h at 42 °C and subsequently for 10 min at 70 °C to terminate the reaction, chilled on ice, and stored at −20 °C. Specific primers were designed to amplify the four p38 isoforms (α, β, γ, δ). In addition, primers with appropriate restriction enzyme sites were designed for cloning the complete coding sequence of Xp38β in the plasmid FTX5 (that contains a Myc tag sequence at the 5′end). *X. laevis* p38α (Xp38α), p38γ (Xp38γ), human p38δ (Hp38δ), cloned in FTX5 vector, and human MKK6-DD (cloned in FTX4 vector, without Myc tag) were obtained from Eusebio Perdiguero (Centre de Regulació Genòmica) and Angel R. Nebreda (Institut de Recerca Biomèdica, Barcelona) and have been described previously (28, 29, 45). MKK6-DD is a constitutively active kinase with the two phosphorylation sites in the activation loop Ser-207 and Thr-211 changed to Glu. *X. tropicalis* p38δ (XTp38δ) was obtained from an I.M.A.G.E cDNA clone (IRBHp990H0820D, Source BioScience), and specific primers were designed for subcloning the coding sequence in FTX5 vector. The primer sequences used are available upon request. PCR reactions were performed in a total volume of 50 μl, with 3 μl of RT product or 10 ng of cDNA plasmid (XTp38δ), 0.2 mM dNTP, 20 μM of each primer (Sigma) and 2.5 units of Pwo Super Yield DNA polymerase (Roche). PCR products were purified, digested with the appropriate restriction enzymes, and cloned in pFTX5 vector.

### Site-directed mutagenesis and *in vitro* transcription

PCR was employed to mutate specific amino acids in the coding sequences. The mutagenesis was performed according to QuikChange Site-Directed Mutagenesis Kit (Stratagene) instruction manual. The primer sequences used for mutagenesis are available upon request.

As described before, Xp38α was obtained from another laboratory. However, after sequencing this construct we detected two consecutive amino acids mutated (E99D and F100P) compared with the published sequence (GenBank, NCBI Reference Sequence: NM_001086831.1). Curiously, the two amino acids mutated (E99 and F100) are codified by an EcoRI restriction site sequence (GAATTC). We assumed that mutation of amino acids EF to DP in Xp38α was a consequence of cloning or subcloning and therefore this construct was corrected by site-directed mutagenesis to obtain the wild-type E99 and F100 amino acids, as reported in GenBank. We also confirmed by RT-PCR and sequencing that endogenous Xp38α mRNA codifies for EF amino acids at positions 99 and 100.

The mutants Xp38α-AF (Thr181 and Tyr183 replaced by Ala and Phe, respectively) and Xp38α-KR (Lys54 replaced by Arg) were also generated. These mutants have been reported as catalytically inactive and can act as a dominant negative in some contexts (46, 47, 48). Analogous mutants (-AF and -KR) were designed for Xp38β, Xp38γ and Hp38δ. Xp38γ-DA (Asp-171 replaced by Ala), a catalytically inactive mutant that inhibits progesterone-induced maturation was a gift of Eusebio Perdiguero (29). Constitutively active p38α (Xp38α-CA) (Asp-177 and Phe-328 replaced by Ala and Ser respectively), and constitutively active p38β (Xp38β-DA/YL) (Asp-176 and Ala-176 replaced by Tyr and Leu, respectively) were generated in our laboratory. It has been reported that these mutants are constitutively active (49, 50).

All cloned genes and mutations generated were confirmed by DNA sequencing. *In vitro* transcriptions of capped RNAs (cRNAs) were obtained by using mMessage mMachine T7 Transcription Kit (Ambion).

### Oocyte injection and hyperosmotic shock treatment

*Xenopus* oocytes (stage VI) were microinjected near their equator with 50 nl (5 ng) of the corresponding cRNAs or H_2_O using a Nanoject II Automatic Nanoliter Injector (Drummond Scientific Company). Injected oocytes were incubated 18 h at 18 °C and poor oocytes were eliminated the next day before treatment. Oocytes were exposed to hyperosmotic shock by transferring them to a new dish containing MBS with 300 mM sorbitol, and pools of 20 oocytes were collected at different times and treated as described below. Previous studies have shown that 300 mM sorbitol induces cytochrome c release and caspase-3 activation between 1 h and 3 h, depending on the experiment (9). In some experiments, oocytes were injected with cytochrome c (0.5 μM final intracellular concentration) from horse heart (c-7752, Sigma) with or without the caspase-3 inhibitor Ac-DEVD-CHO (Molecular Probes) at 0.1 or 1 μM final intracellular concentration and collected 1 h later. As a control, some oocytes were injected only with MBS, the solvent used for cytochrome c and Ac-DEVD-CHO.

### Oocyte lysis and Western blot analysis

Pools of 20 oocytes were lysed by pipetting up and down in 200 μl of ice-cold extraction buffer (0.25 M sucrose, 0.1 M NaCl, 2.5 mM MgCl_2_, 20 mM HEPES, pH 7.2) containing 1 mM EDTA, 1 mM EGTA, protease inhibitors (10 μg/ml leupeptin, 1 mM PMSF, 10 μg/ml aprotinin) and phosphatase inhibitors (50 mM β-glycerolphosphate, 50 mM sodium fluoride, 1 mM sodium orthovanadate, 5 mM sodium pyrophosphate). Samples were clarified by centrifugation at 14.500 rpm for 5 min and supernatants (cytosolic fraction) were collected and processed for immunoblotting or caspase assay as described below. The whole supernatants were denatured with Sample Buffer (50 mM Tris HCl, pH 6.8, SDS 2%, 100 mM dithiothreitol, 10% glycerol) and subjected to 10% or 15% SDS/PAGE and transferred to Immobilon-P membranes (Millipore). 16 μl of cell extract was loaded in each well (equivalent to 32 μg protein). In our experience, more errors are made trying to load similar amounts of protein measured with a colorimetric method. The uniformity of samples loading was verified by Ponceau (Sigma) staining of the blots. Membranes were blocked for 1 h with 5% dried skimmed milk in TBST (50 mM Tris, 150 mM NaCl, 100 mM KCl, pH 7.4, and 0.1% Tween 20) and then incubated with the following antibodies: anti-AMPKα (#2532, Cell Signaling), anti-pp38 (Thr180/Tyr182) (#9211, Cell Signaling), monoclonal anti-Myc (M4439, clone 9E10, Sigma), monoclonal anti-β-actin (A1978, Sigma), and anti-Cytochrome c (556,432, BD Pharmingen). Antibody binding was detected with horseradish peroxidase–coupled secondary antibody and the enhanced chemiluminescence (ECL) detection kit (Amersham Biosciences). Blots were quantified with Image J and the ratio pp38/AMPK, pp38/Myc or cytochrome c (CC)/AMPK represented.

### Assay for DEVDase activity

Caspase-3 activity was measured in terms of DEVDase activity assay in 96 cells opaque plates (OptiPlate, Ref. 6,005,270, PerkinElmer). 25 μl cytosolic fraction (corresponding to 2.5 oocytes) were diluted 1:1 with 25 μl lysis buffer and assayed with 50 μl 2 × Reaction Buffer (20% glycerol, 40 mM Hepes, 4 mM DTT, pH 7.5) containing 200 μM of synthetic peptide Z-DEVD-AMC (Peptide Institute, Inc.). Fluorescence at 360 nm for excitation and at 460 nm for emission was measured after incubation of the samples for 60 min at 37 °C. Caspase-3 activity was determined as the concentration of fluorescent AMC formation from Z-DEVD-AMC substrate and represented as arbitrary units (AU) of caspase-3 activity, giving value 1 to non-treated oocytes injected with H_2_O.

### Statistical analysis

Data are expressed as means ± SD. Statistical analysis was performed with the GraphPad Prism 8.0.1 program. One-way ANOVA with a Dunnett Multiple Comparison Test was used in oocytes microinjected with different cRNAs comparing all columns *versus* water injected oocytes. Uncorrected Fisher’s LSD test was used when several groups injected with cytochrome c were compared with their respective controls (non-injected). Values of *p* < 0.05 were considered statistically significant.

## Data availability

All the relevant data are contained within this article. Primer sequences used to generate the different constructs are available upon request to the corresponding author (J. M. L.).

## Supporting information

This article contains supporting information.

## Conflict of interest

The authors declare that they have no conflicts of interest with the contents of this article.
