# Supplementary material for: p38α and p38β regulate osmostress-induced apoptosis
Source: J Biol Chem. 2024 Dec 7;301(1):108061. doi: 10.1016/j.jbc.2024.108061 (PMC11757794; doi:10.1016/j.jbc.2024.108061)
Supplement: Supporting Information [file mmc1.pdf]

Supporting information for:

p38α and p38β regulate osmostress-induced apoptosis

Running title: Role of p38 isoforms in hyperosmotic shock

Nabil Ben Messaoud and José M. López*

Institut de Neurociències, Departament de Bioquímica i Biologia Molecular, Unitat de Bioquímica, Facultad de Medicina, Universitat Autònoma de Barcelona, 08193 Cerdanyola del Vallès, Barcelona, Spain.

Contact information:

* Corresponding author

E-mail: josemanuel.lopez@uab.cat

ORCID: 0000-0002-4104-6262

Figure S1

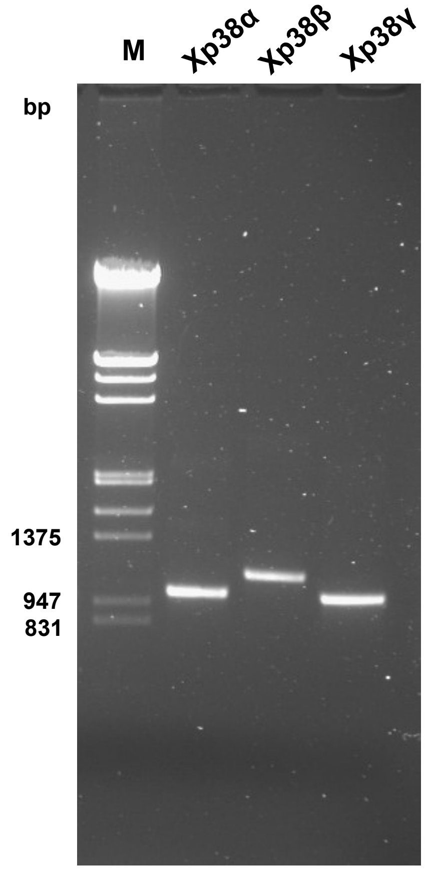

Figure S1. p38α (MAPK14), p38β (MAPK11), and p38γ (MAPK12) are expressed in *Xenopus laevis* oocytes. Total RNA isolated from oocytes (stage VI) was subjected to RT-PCR using specific primers for each subunit. 50 ng of DNA product was loaded in the gel. Three p38 subunits (α, β, γ) were detected. The left lane (M) corresponds to a DNA marker.

Figure S2

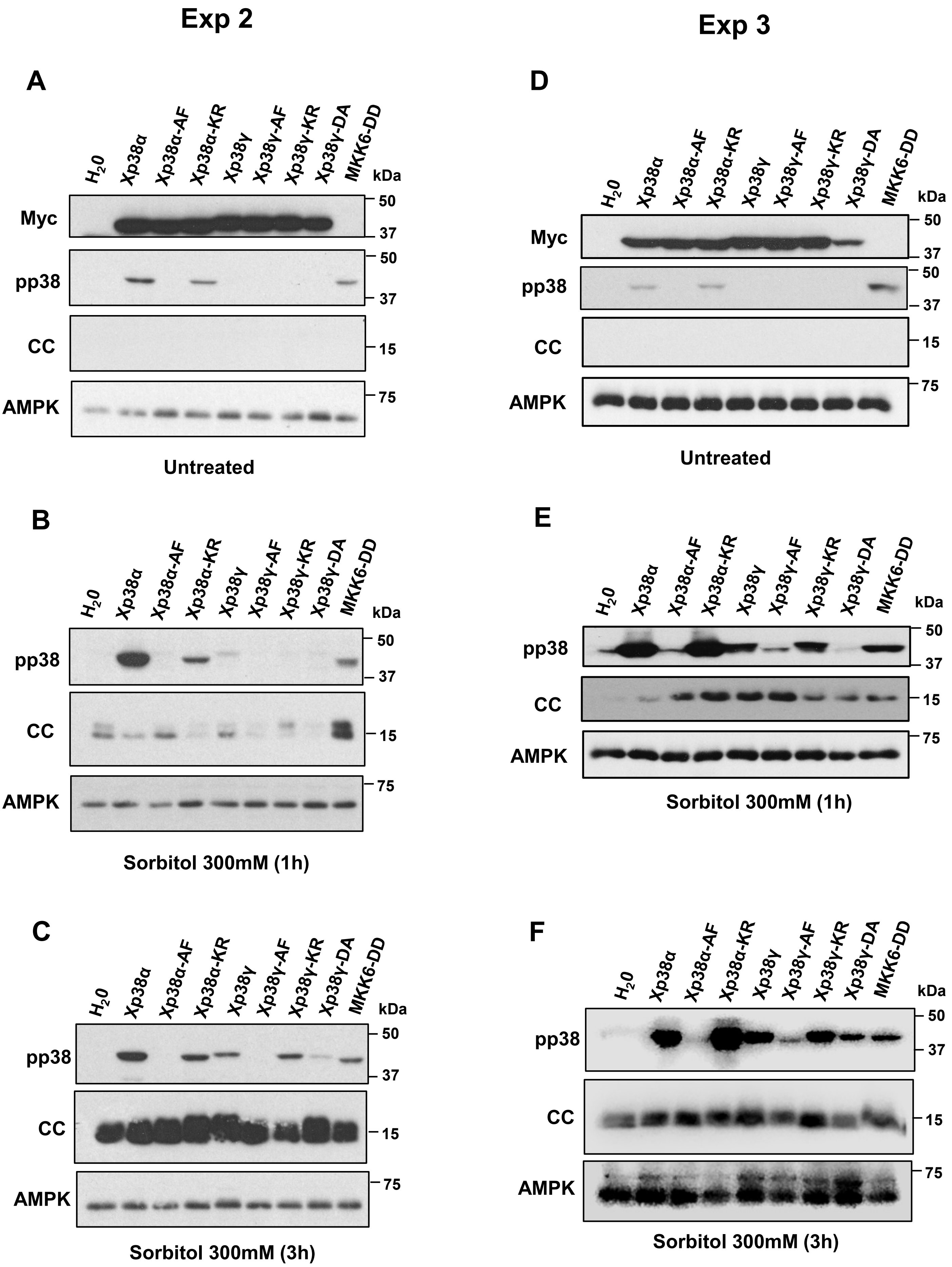

Exp 2
Exp 3
A
H20 Xp38α Xp38α-AF Xp38α-KR Xp38γ Xp38γ-AF Xp38γ-KR Xp38γ-DA MKK6-DD
kDa
Myc
pp38
CC
AMPK
50
37
50
37
15
75
Untreated
B
H20 Xp38α Xp38α-AF Xp38α-KR Xp38γ Xp38γ-AF Xp38γ-KR Xp38γ-DA MKK6-DD
kDa
pp38
CC
AMPK
50
37
15
75
Sorbitol 300mM (1h)
C
H20 Xp38α Xp38α-AF Xp38α-KR Xp38γ Xp38γ-AF Xp38γ-KR Xp38γ-DA MKK6-DD
kDa
pp38
CC
AMPK
50
37
15
75
Sorbitol 300mM (3h)
D
H20 Xp38α Xp38α-AF Xp38α-KR Xp38γ Xp38γ-AF Xp38γ-KR Xp38γ-DA MKK6-DD
kDa
Myc
pp38
CC
AMPK
50
37
50
37
15
75
Untreated
E
H20 Xp38α Xp38α-AF Xp38α-KR Xp38γ Xp38γ-AF Xp38γ-KR Xp38γ-DA MKK6-DD
kDa
pp38
CC
AMPK
50
37
15
75
Sorbitol 300mM (1h)
F
H20 Xp38α Xp38α-AF Xp38α-KR Xp38γ Xp38γ-AF Xp38γ-KR Xp38γ-DA MKK6-DD
kDa
pp38
CC
AMPK
50
37
15
75
Sorbitol 300mM (3h)

Figure S2. Xp38α and Xp38γ are activated by hyperosmotic shock. Expression and phosphorylation of Xp38α, Xp38γ, and their mutants. Two additional experiments are presented with conditions identical to those described in Figure 1: experiment 2 (A, B, C) and experiment 3 (D, E, F). Oocytes were injected with 50 nL of H_2O or cRNAs (5 ng in 50 nL) Xp38α, Xp38α-AF, Xp38α-KR, Xp38γ, Xp38γ-DA, or MKK6-DD and 18 h later exposed to osmotic shock (300 mM sorbitol) for 1 h, 3 h, or non-treated. Expression of p38 isoforms was confirmed with Myc antibodies (A and D). MKK6-DD is a constitutively active mutant without Myc tag. pp38 and cytosolic cytochrome c (CC) levels were analyzed by Western blot and AMPK was used as a loading control.

Figure S3

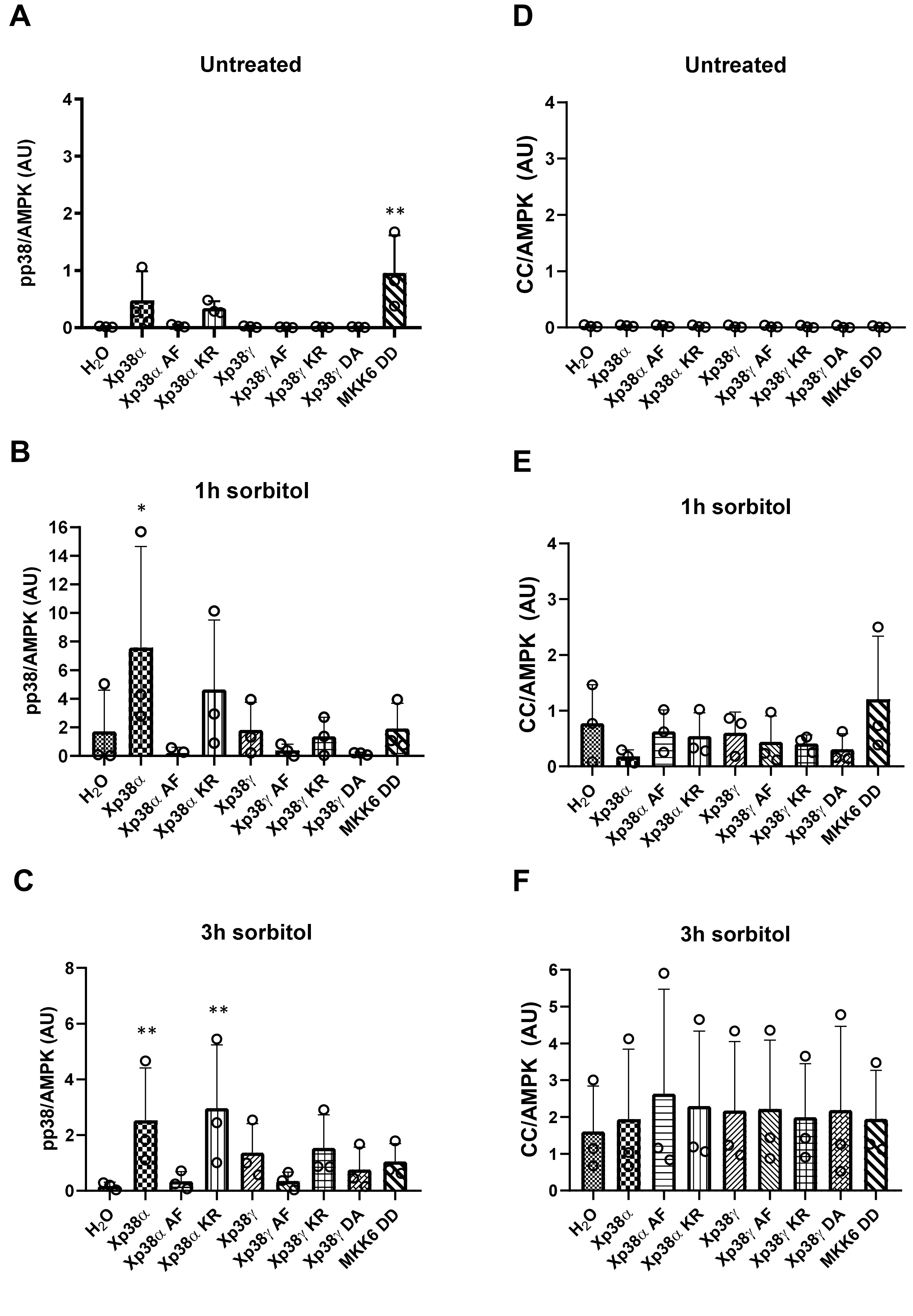

Figure S3. Quantification of Western blots from Figures 1 and S2. Blots were quantified with Image J and the ratio pp38/AMPK (A, B, C) and CC/AMPK (D, E, F) represented. Results are the mean ± SD of three independent experiments. *p<0.05, **p<0.01 compared to water-injected oocytes (ANOVA and Dunnett's test).

Figure S4

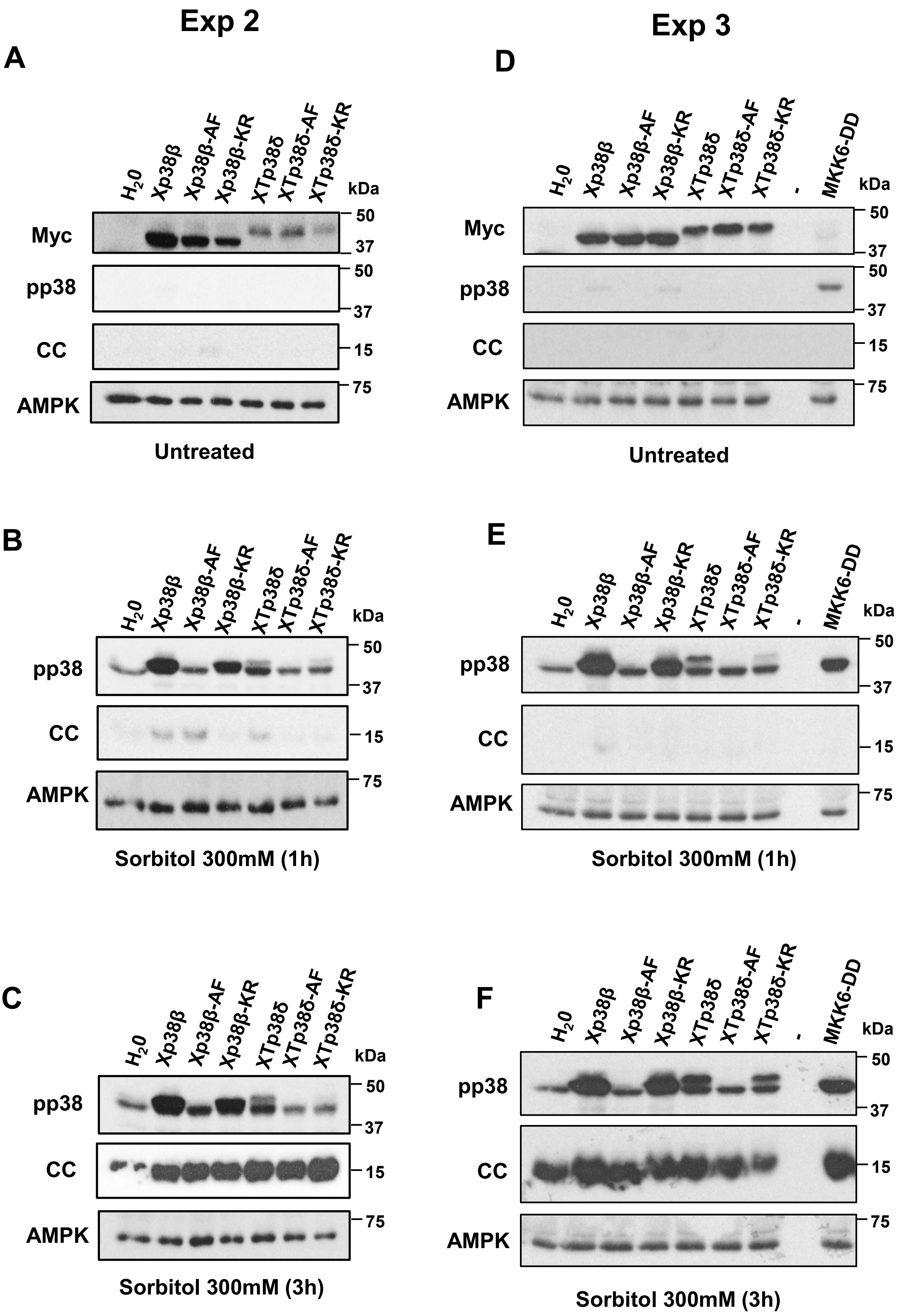
Exp 2
Exp 3
A
H_2O
Xp38β
Xp38β-AF
Xp38β-KR
XTp38δ
XTp38δ-AF
XTp38δ-KR
kDa
Myc
pp38
CC
AMPK
50
37
15
75
Untreated
D
-
MKK6-DD
Untreated
B
Sorbitol 300mM (1h)
E
Sorbitol 300mM (1h)
C
Sorbitol 300mM (3h)
F
Sorbitol 300mM (3h)

Figure S4. Xp38β is activated by hyperosmotic shock. Expression and phosphorylation of p38 isoforms and mutants. Two additional experiments are presented with conditions identical to those described in Figure 2: experiment 2 (A, B, C) and experiment 3 (D, E, F). Oocytes were injected with 50 nL of H_2O or cRNAs (5 ng in 50 nL) Xp38β, Xp38β-AF, Xp38β-KR, Xp38δ, Xp38δ-AF, Xp38δ-KR, or MKK6-DD and 18 h later exposed to osmotic shock (300 mM sorbitol) for 1 h, 3 h, or non-treated. Expression of p38 isoforms was confirmed with Myc antibodies (A and D). pp38 and cytosolic cytochrome c (CC) levels were analyzed by Western blot and AMPK was used as a loading control.

Figure S5

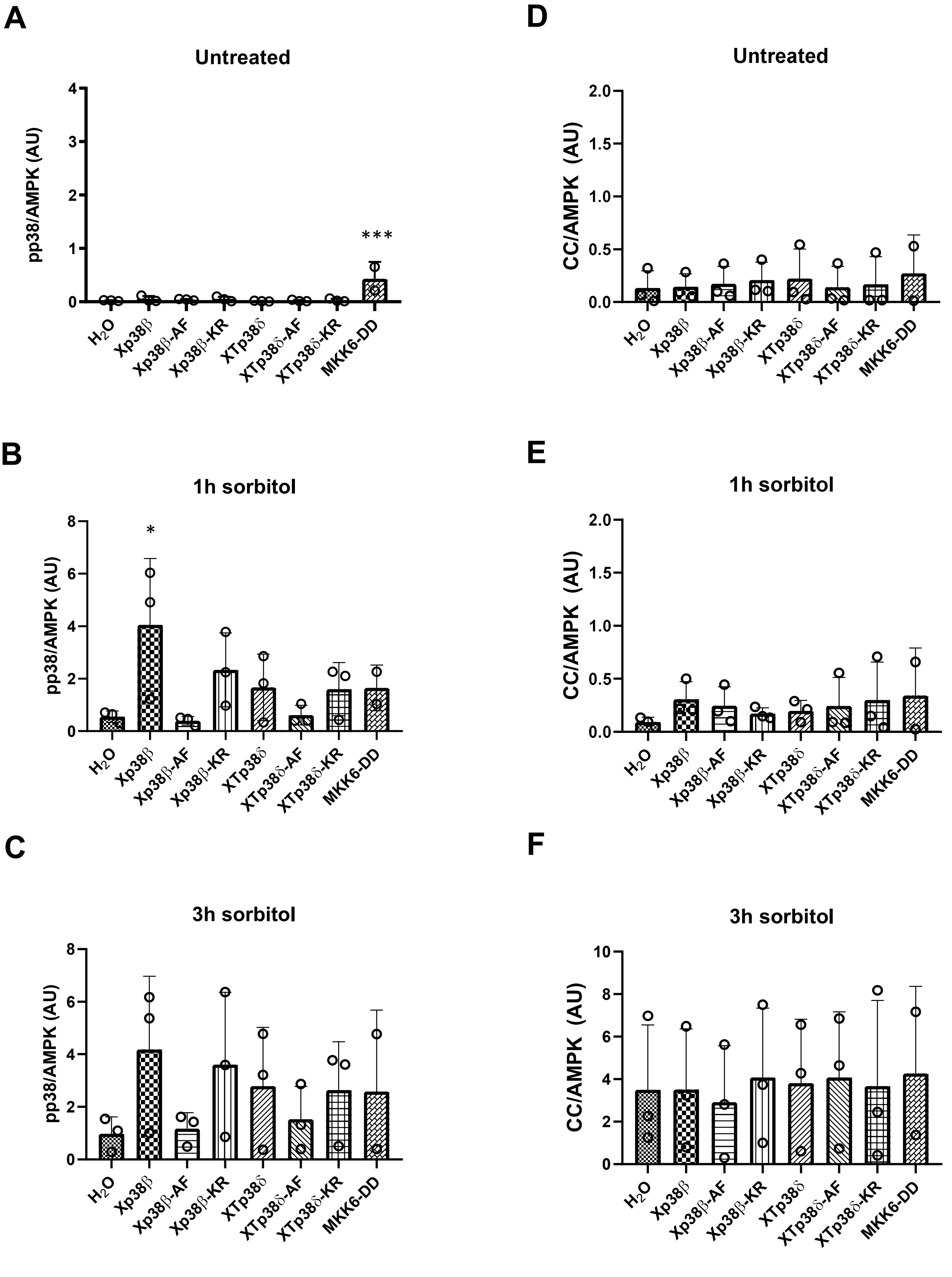

Figure S5. Quantification of Western blots from Figures 2 and S4. Blots were quantified with Image J and the ratio pp38/AMPK (A, B, C) and CC/AMPK (D, E, F) represented. Results are the mean ± SD of three independent experiments. *$p<0.05$, ***$p<0.001$ compared to water-injected oocytes (ANOVA and Dunnett's test).

Figure S6

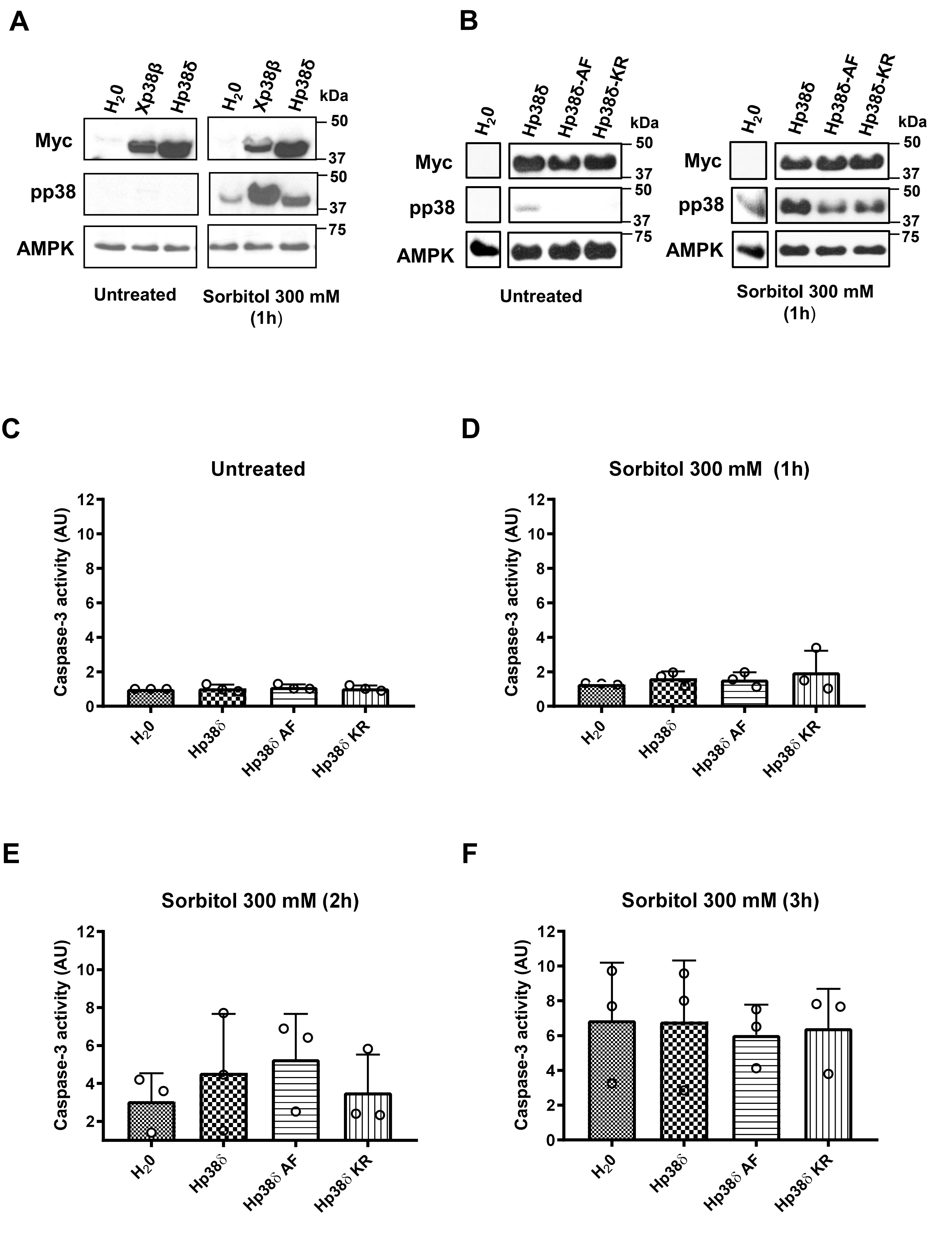
A
H20
Xp38β
Hp38δ
kDa
Myc
pp38
AMPK
50
37
75
Untreated
Sorbitol 300 mM (1h)
B
Hp38δ-AF
Hp38δ-KR
C
Untreated
Caspase-3 activity (AU)
Hp38δ AF
Hp38δ KR
D
Sorbitol 300 mM (1h)
E
Sorbitol 300 mM (2h)
F
Sorbitol 300 mM (3h)

Figure S6. Expression and phosphorylation of human p38δ (Hp38δ) in *Xenopus* oocytes. (A) *Xenopus* oocytes were injected with H_2O or cRNAs Xp38β, Hp38δ, and 18 h later exposed to 300 mM sorbitol for 1 h, or non-treated. Pools of 20 oocytes were collected and analyzed by Western blot to measure Myc, pp38, and AMPK (loading control). Note that expression of Hp38δ is higher that Xp38β (Myc western) but phosphorylation of Hp38δ is lower that Xp38β (pp38 western). (B) Expression of Hp38δ or the mutants Hp38δ-AF and Hp38δ-KR in *Xenopus* oocytes untreated or treated with 300 mM sorbitol for 1 h. (C-F) Oocytes were injected with 50 nl of H_2O or cRNAs (5 ng in 50 nl) Hp38δ, Hp38δ-AF, Hp38δ-KR and 18 h later exposed to 300 mM sorbitol for different times (1 h, 2 h, and 3 h) or non-treated. Caspase-3 activity was determined in all the conditions, giving value 1 to non-treated water-injected oocytes. Results are the mean ± SD of 3 independent experiments. Expression of Hp38δ or its mutants do not modify hyperosmotic shock-induced apoptosis (ANOVA and Dunnett's test).

Figure S7

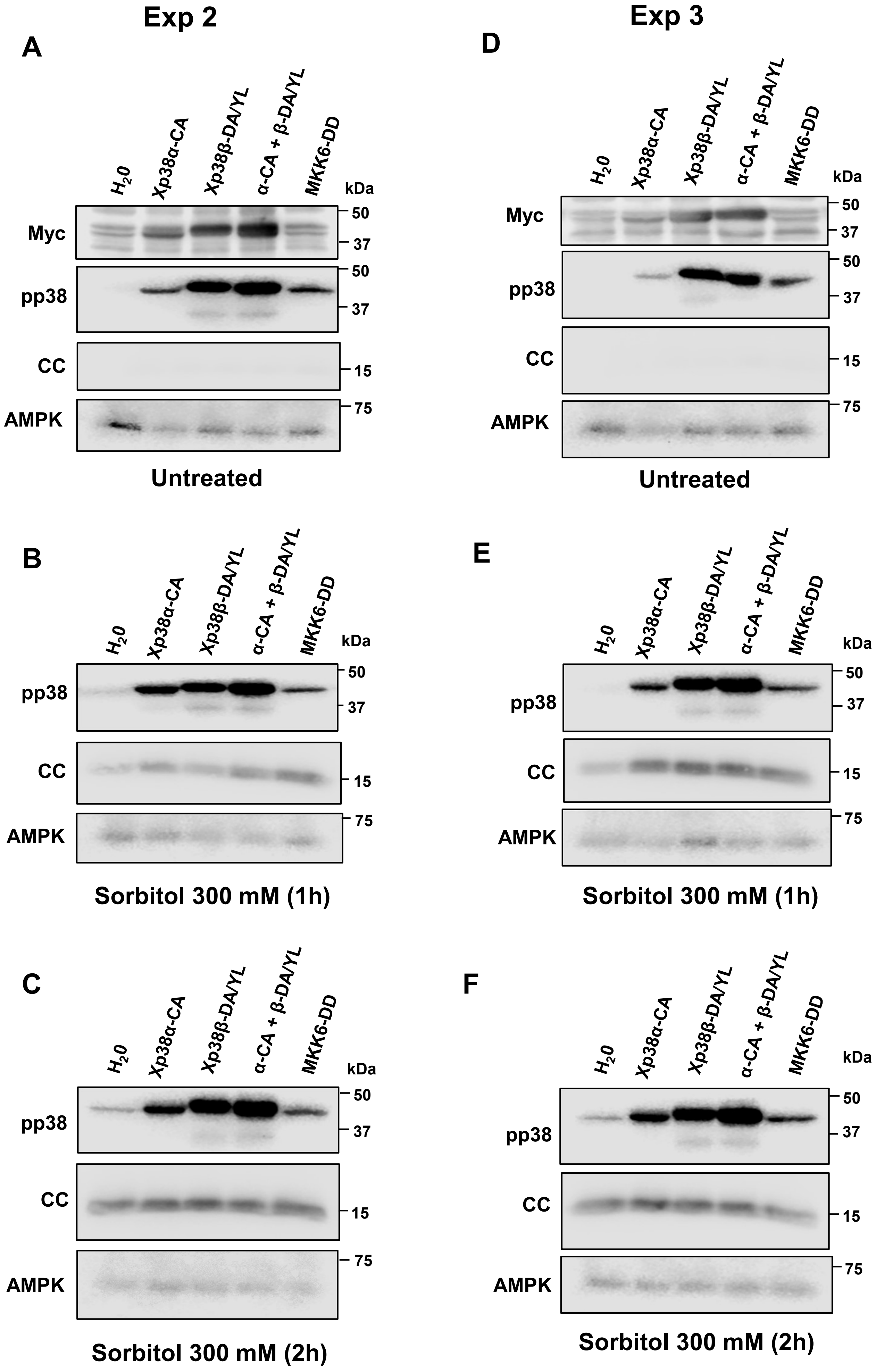
Exp 2
Exp 3
A
H20
Xp38α-CA
Xp38β-DA/YL
α-CA + β-DA/YL
MKK6-DD
kDa
Myc
pp38
CC
AMPK
50
37
15
75
Untreated
D
B
Sorbitol 300 mM (1h)
E
C
Sorbitol 300 mM (2h)
F

Figure S7. Constitutively active Xp38α (CA) or Xp38β (DA/YL) expression accelerates hyperosmotic shock-induced apoptosis. Two additional experiments are presented with conditions identical to those described in Figure 3: experiment 2 (A, B, C) and experiment 3 (D, E, F). Oocytes were injected with 50 nL of H_2O or cRNAs (5 ng in 50 nL) Xp38α-CA, Xp38β-DA/YL, a combination of both, or MKK6-DD and 18 h later exposed to osmotic shock (300 mM sorbitol) for 1 h, 2 h, or non-treated. Expression of p38 mutants was confirmed with Myc antibodies (A and D). pp38 and cytosolic cytochrome c (CC) levels were analyzed by Western blot and AMPK was used as a loading control.

Figure S8

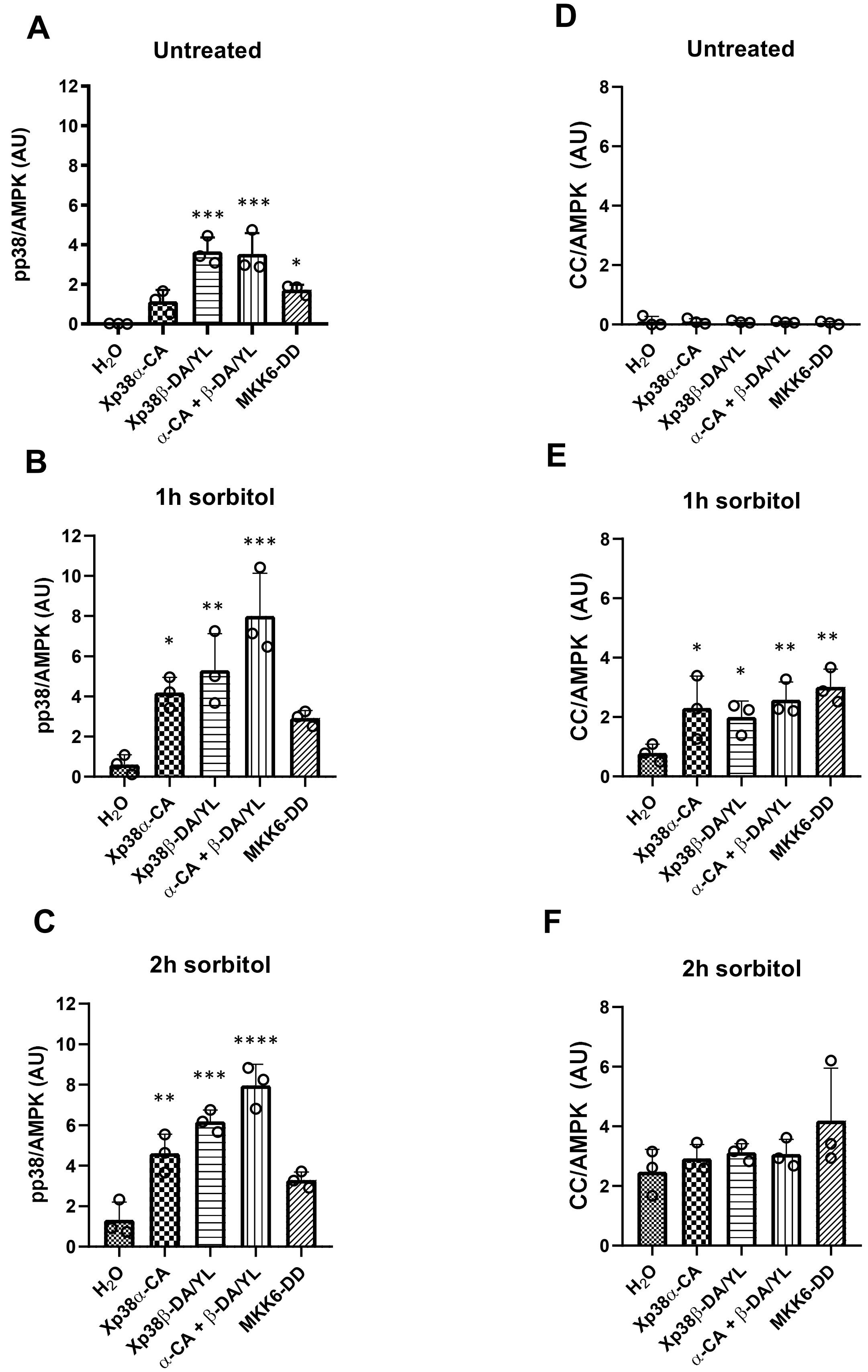

Figure S8. Quantification of Western blots from Figures 3 and S7. Blots were quantified with Image J and the ratio pp38/AMPK (A, B, C) and CC/AMPK (D, E, F) represented. Results are the mean ± SD of three independent experiments. *p<0.05, **p<0.01, ***p<0.001 ****p<0.0001 compared to water-injected oocytes (ANOVA and Dunnett's test).

Figure S9

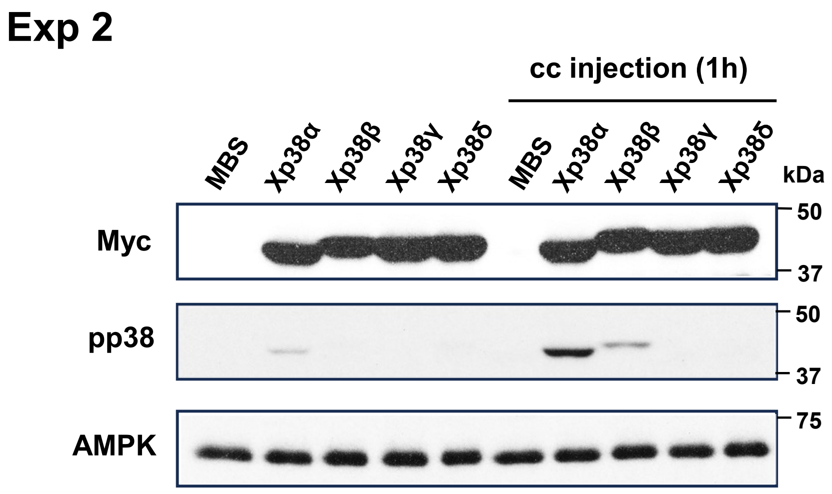

Figure S9. Cytochrome c injection in *Xenopus* oocytes induces caspase-3 activation and p38α and p38β phosphorylation. An additional experiment is presented with conditions identical to those described in Figure 4. Oocytes were injected with 50 nl of MBS or cRNAs (5ng in 50 nl), Xp38α, Xp38β, Xp38γ, or Xp38δ and 18 h later injected with MBS or horse cytochrome c dissolved in MBS (CC) (0.5 μM final concentration) and pools of 20 oocytes were lysed 1 h later to analyze Myc, pp38, and AMPK by Western blot.
